# The complete mitochondrial genome of *Ahamus yushuensis* Chu et Wang 1985 (Lepidoptera: Hepialidae) and phylogenetic analysis

**DOI:** 10.1080/23802359.2022.2116950

**Published:** 2022-09-08

**Authors:** Xiuzhang Li, Yuling Li

**Affiliations:** State Key Laboratory of Plateau Ecology and Agriculture, Qinghai Academy of Animal and Veterinary Science, Qinghai University, Xining, China

**Keywords:** Hepialidae, mitochondrial DNA, phylogenetic analysis

## Abstract

The complete mitochondrial genome (mitogenome) of *Ahamus yushuensis* was determined in this study. This mitogenome is 15,336 bp and encodes 37 mitochondrial genes, including 13 protein-coding genes (PCGs), 22 transfer RNA genes (tRNAs), and two ribosomal RNA genes (*rrnL* and *rrnS*). The *A. yushuensis* mitogenome has an A + T content of 82.2% and presents a positive AT-skew (0.052) and a negative GC-skew (−0.236). Twelve PCGs start with a typical ATN codon, whereas a single PCG uses CGA (*coxI*) as the initial codon. The maximum likelihood phylogenetic analysis based on the concatenated nucleotide sequences of 13 PCGs strongly supported the monophyletic relationship of *A. yushuensis* to the clade of *Thitarodes damxungensis* and *A. yunnanensis.*

*Ahamus yushuensis* Chu et Wang 1985 (Lepidoptera: Hepialidae) is a species of moth of the family Hepialidae, which is the main host of *Ophiocordyceps sinensis* (=*Cordyceps sinensis*) (Wang and Yao 2011; Li et al. 2017), a species of parasite ascomycete fungi with a high commercial value given its importance for traditional Chinese medicine (Zhang et al. 2012; Cheng et al. 2016; Jin et al. 2020). For a deeper knowledge of this species, here we reported the sequences of complete mitochondrial genome of *A. yushuensis* firstly.

The sample was collected from Yushu Tibetan Autonomous Prefecture of Qinghai provience, China (N: 33°01′, E: 96°48′; Elevation: 4361.6), in August 2020. Samples have been deposited in Qinghai Academy of Animal and Veterinary Sciences, Qinghai University, Xining, China (ZQHU20-YS31, Xiuzhang Li, xiuzhang@qhu.edc.cn). There was no endangered or protected species involved in the study, and no specific permissions were required for the sample. Additionally, this study was supported by grants from Major science and technology projects of Qinghai Province (2021-SF-A4). Total genomic DNA was extracted from a single specimen using a DNeasy Tissue Kit (Qiagen, German). The mitogenome sequencing of *H. yushuensis* was performed on an Illumina NovaSeq Platform (Illumina, San Diego, CA), and then the initial annotation of the mitogenome was carried out with MITObim v1.9 and SOAPdenovo v2.04 (Xie et al. 2014). The gene boundaries were verified by MITOS2 (Alexander et al. 2019).

The sequence with annotated features has been deposited in GenBank under Accession No. MZ748305. The *A. yushuensis* complete mitogenome sequence length was 15,336 bp, encoding13 protein-coding genes (PCGs), two rRNA genes, 22 tRNA genes, and a non-coding control region (D-loop). The order and orientation of the mitochondrial genes was identical to the inferred ancestral arrangement of insects (Boore 1999). Gene overlaps were found at eight gene junctions and involved a total of 32 bp, with the longest overlap (8 bp) between *trnW* and *trnC*.

The nucleotide composition of the *A. yushuensis* mitogenome was significantly biased toward A and T, with an A + T content of 82.2% (A = 41.3%, C = 10.4%, G = 7.5%, T = 40.9%) showed a positive AT-skew (0.052) and a negative GC-skew (–0.236) on the J-strand. The lengths of *A. yushuensis* small subunit ribosomal RNA and large subunit ribosomal RNA were 773 bp and 1273 bp, respectively. The control region (503 bp) with 88.5% A + T content was placed between 12S rRNA and tRNA-Ile. For start codons, most PCGs started with standard codon ATN, except for *cox1* initiated with CGA. Twelve genes terminated with TAA, *cox2* ended with an incomplete T—.

The maximum likelihood (ML) phylogenetic tree was built based on 13 PCG of 26 Lepidoptera species complete mitochondrial genome, and *Drosophila melanogaster* as the outgroup. We used PartitionFinder to find the corresponding nucleotide substitution (Lanfear et al. 2017). A maximum likelihood tree was built using IQ-TREE (Nguyen et al. 2015). Phylogenetic relationships among nine families within Lepidoptera were recovered as can be seen in Figure 1. Phylogenetic analysis reveals a well-supported clade including *A. yushuensis*, *Thitarodes damxungensis* and *Ahamus yunnanensis*, suggesting that the taxonomy of *Thitarodes* species should be revised (Figure 1).

**Figure 1.**
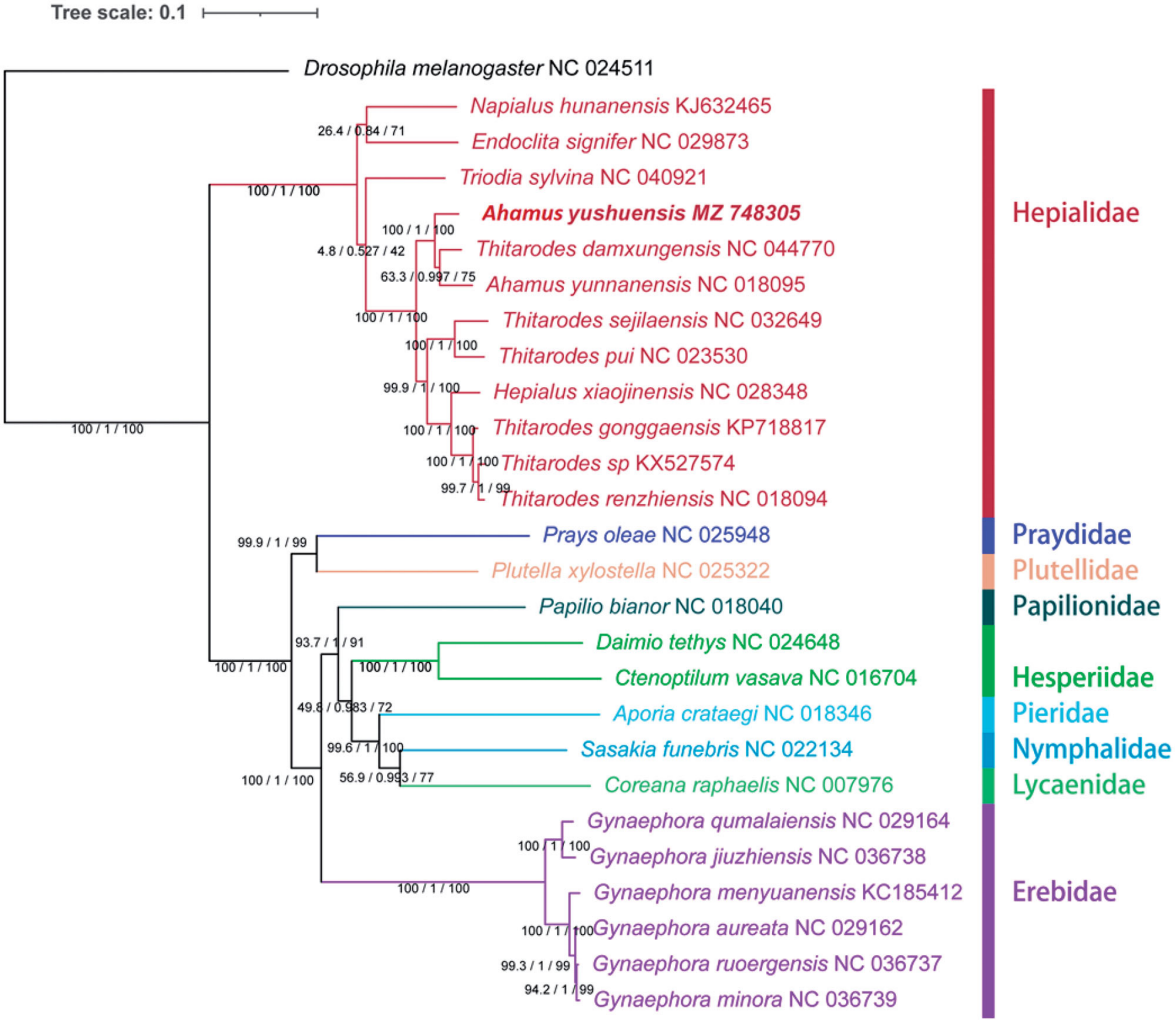
Mitochondrial phylogeny of 26 Lepidoptera species based on the concatenated nucleotide sequences of 13 mitochondrial PCGs. *Drosophila melanogaster* was used as an outgroup. GenBank accession numbers of each species are listed in the tree.

## Data Availability

The genome sequence data that support the findings of this study are openly available in GenBank of NCBI at (https://www.ncbi.nlm.nih.gov/) under accession No. MZ748305 (https://www.ncbi.nlm.nih.gov/nuccore/MZ748305). The associated BioProject, SRA, and Bio-Sample numbers are PRJNA796659 (https://www.ncbi.nlm.nih.gov/bioproject/PRJNA796659/), SRA: SRR17593536 (https://www.ncbi.nlm.nih.gov/sra/SRR17593536/), and SAMN24906808 (https://www.ncbi.nlm.nih.gov/biosample/?term=SAMN24906808) respectively.
